# Effects of Horizontal and Vertical Vector Resistance Training on Swim Start Performance: An Eight-Week Intervention in Division One Collegiate Swimmers in Taiwan

**DOI:** 10.3390/jfmk10030236

**Published:** 2025-06-22

**Authors:** Jyun-Ru Chen, Yu-Lin Ning, Ting-Yao An, Yi-Lin Tsai, Kuo-Wei Tseng, Chi-Chieh Hsu

**Affiliations:** 1 Institute of Sports Science, University of Taipei, Taipei 111036, Taiwan; d11131002@go.utaipei.edu.tw; 2 Department of Exercise and Health Science, University of Taipei, Taipei 111036, Taiwan; fossil0405@yahoo.com.tw; 3 Department of Aquatic Sports, University of Taipei, Taipei 111036, Taiwan; andy8072380723@gmail.com (T.-Y.A.); hungjia2008@gmail.com (Y.-L.T.); 4 Department of Martial Arts, University of Taipei, Taipei 111036, Taiwan; tpec010@gmaill.com; 5 Graduate Institute of Sports Training, University of Taipei, Taipei 111036, Taiwan

**Keywords:** resistance training, force-vector training, training transfer

## Abstract

**Background**: This study aimed to compare the effects of an eight-week horizontal versus vertical vector resistance training program on swim start performance and lower-limb neuromuscular function in competitive swimmers. **Methods**: A total of 16 collegiate swimmers (14 males and 2 females; height: 176.3 ± 10 cm; body mass: 68.8 ± 10.3 kg; age: 20.5 ± 2.3 year) were assigned to either a horizontal vector training (HOR) or a vertical vector training (VER) group and completed an eight-week training program. Pre- and post-intervention assessments included flight time, flight distance, underwater speed, 15 m swim speed, 25 m swim speed, and force–time metrics within both concentric and eccentric phases of the countermovement jump and squat jumps. **Results**: No group or interaction effects were observed. However, time effects were found for flight distance (↑ 4.1–5.5%), flight time (↑ 6.2–12%), 15 m swim speed (↑ 0.3–0.7%), and jump performance. The HOR showed more favorable within-group trends in regards to swim start performance and concentric performance of countermovement and squat jumps. Moderate correlations (r = 0.450–0.476) were found between changes in concentric jump variables and 15 m swim speed. **Conclusions**: These results suggest that both vertical and horizontal vector resistance training can improve lower-limb neuromuscular performance and swim start performance.

## 1. Introduction

Short-distance swimming performance, especially in 50 m freestyle events, is greatly influenced by the swimming start [1]. Recent Olympic Games have shown that outcomes in 50 m events are often determined by mere hundredths of a second, emphasizing the importance of optimizing the start technique through appropriate training. According to deterministic models of swimming performance, total race time is determined by a combination of biomechanical, technical, and energetic factors that operate across distinct race phases—including the start, stroke, and finish [2]. Within this framework, the start phase can be further subdivided into the block phase, flight and entry phase (e.g., flight distance and time), and the underwater phase, which typically covers the first 15 m of the freestyle race [3,4,5,6,7,8]. As Thng et al. [3] emphasized, a successful start requires swimmers to apply force rapidly on the starting block and to maximize take-off velocity in the intended direction, enabling effective transfer of momentum into the underwater phase [4,6]. This ability depends largely on the combination of lower-limb explosive strength and precise force orientation.

Previous research has shown that the kick-start, now the most widely used start technique in competitive swimming, generates greater horizontal impulse and take-off velocity than the traditional grab starts, while also reducing block time [9]. Follow-up studies have identified horizontal take-off velocity and average power output as key determinants of 15 m swim start performance, with peak horizontal force from both the front and rear legs recognized as important predictors [4]. Notably, it has been further reported that female swimmers tend to rely more heavily on horizontal force production during the start phase compared to male swimmers, suggesting potential sex-based differences in start mechanics [10].

Force-vector theory is a training concept that highlights the importance of aligning the direction of force application with the specific demands of a given sport movement [11]. This principle has been increasingly adopted in various sports disciplines in recent years [12]. In the context of swimming, vertical resistance training—including squats, Olympic lifts, and plyometric exercises—has traditionally been used to enhance lower-body power with the aim of improving jump distance, take-off velocity, and reaction time [13,14,15]. In contrast, horizontal resistance training—including sled pushes, resisted sprints, horizontal jumps, and hip thrusts—has been more commonly employed in sports like track and field or soccer, where horizontal propulsion and sprinting ability are critical, showing greater transfer to these sport-specific tasks [16,17,18].

Investigating the long-term effects of different force-vector-oriented training strategies could help clarify their performance transferability and inform the design of more effective training interventions. Despite these findings, empirical research on the application of horizontal resistance training in swimming remains limited [19,20]. To date, only one recent study has investigated its short-term effects, specifically in relation to post-activation performance enhancement (PAPE) [19]. Oğul et al. examined the acute effects of a single set of three repetitions at 80% 1RM during warm-up and found that hip thrust training improved the flight phase of the swim start, whereas squats did not yield the same benefit. Regarding long-term training adaptations, only one study has directly compared the effects of horizontal and vertical resistance training on swim start performance [20]. Furthermore, how these two training orientations affect jump performance and swimming propulsion—especially within the critical first 15 m of the race—remains underinvestigated.

Therefore, the purpose of this study was to compare the effects of eight weeks of vertical and horizontal resistance training on swim start performance, with a particular focus on 15 m swim speed, associated performance indicators, and 25 m swim performance. Although both training modalities are expected to improve take-off ability, we hypothesize that horizontal resistance training (HOR) will yield greater improvements in 15 m swim speed due to its closer alignment with the direction of swim propulsion. Additionally, this study aims to explore whether improvements in jump performance are associated with swim performance gains and whether these improvements are consistent across different jump-related metrics.

## 2. Materials and Methods

### 2.1. Experimental Design

**Figure 1:**
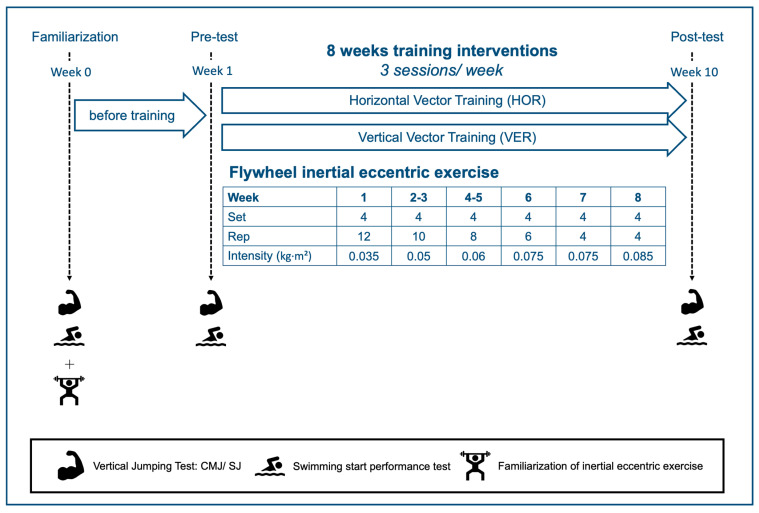
Study design. Participants completed one familiarization session in Week 0 to learn the inertial eccentric exercise using a flywheel device. Pre-tests were conducted in Week 1, followed by an 8-week training program (three sessions/week) comprising either horizontal vector training (HOR) or vertical vector training (VER) using flywheel-based resistance. Training intensity was progressively increased each week, based on the moment of inertia (kg·m^2^). Post-tests were conducted in Week 10. Vertical jumping tests (CMJ/SJ) and swimming start performance tests were conducted during both pre- and post-intervention.

This study was a matched, parallel trial conducted over a 10-week period, including pre- and post-testing phases, along with an eight-week training intervention (Figure 1). Participants completed three laboratory visits throughout the study. During the first visit, which took place one week before the official experiment, the participants were introduced to the study procedures and assessment protocols and were familiarized with the inertial eccentric training movements. This ensured that they understood the testing process and could perform the required exercises correctly. The second visit occurred during the pre-test week, where participants underwent baseline assessments, including body composition measurements, vertical jump tests, and swim start performance tests. Following the pre-tests, participants were stratified based on their 15 m swim start performance to ensure a balanced distribution of swimming ability. The participants were pair-matched based on their 15 m swim speeds and then randomly assigned within each pair to one of the two training groups: vertical vector training (VER) or horizontal vector training (HOR). The training intervention lasted for eight weeks, with sessions conducted three times per week immediately after in-water swimming training. Each session took place from 17:00 to 18:00 and was conducted using an inertial eccentric training device (kBox Pro4, kBox, Stockholm, Sweden) in the weightroom. Throughout the intervention period, participants maintained their regular swimming and dry-land training, as the study overlapped with their sport-specific preparation phase and pre-competition phase. Following the completion of the training intervention, participants returned for a third laboratory visit to undergo post-tests, replicating the same assessment protocols as those for the pre-test.

### 2.2. Participants

This study recruited 20 competitive swimmers who had participated in national-level competitions in Taiwan. The participants were required to meet the following inclusion criteria: (1) must be the collegiate division-one swimmers aged between 18 and 26 years, (2) had at least four years of swim training experience under a professional coach, (3) had undergone strength training for over a year under the supervision of an NSCA-CSCS certified coach, (4) had incurred no major musculoskeletal injuries, cardiovascular diseases, diabetes, or other internal medical conditions or surgeries within the past six months, and (5) agreed to abstain from alcohol and caffeine for at least 24 h before the experiment. At the beginning of the study, the participants underwent body composition assessments, which recorded age, height, body mass, body fat percentage, and skeletal muscle mass. An athletic trainer conducted health screenings, confirming the absence of relevant sports injuries or medical conditions. All participants were informed about the study’s benefits and potential risks before providing their written informed consent. This study was conducted in accordance with the Declaration of Helsinki and approved by the institutional review board of the University of Taipei (Taipei, Taiwan; approval no.: IRB-2020-054). Due to personal reasons, four participants did not complete the post-test and withdrew from the study. The basic characteristics of the participants are presented in Table 1.

### 2.3. Training Intervention

The participants were divided into two training groups: VER and the HOR, both focusing on lower-body exercises. The VER group performed half squats as the primary training exercise, while the HOR group performed hip thrusts. The training intervention lasted eight consecutive weeks, with three sessions per week, totaling 24 training sessions. Each force vector training session was performed immediately after the in-water swimming training, between 17:00 and 18:00. The training program followed a linear periodization model, gradually increasing intensity in alignment with the swim team’s training cycle. Participants performed 4 sets of 12 repetitions at 0.035 kg·m^2^ in Week 1, 4 sets of 10 repetitions at 0.050 kg·m^2^ in Weeks 2–3, 4 sets of 8 repetitions at 0.060 kg·m^2^ in Weeks 4–5, 4 sets of 6 repetitions at 0.075 kg·m^2^ in Week 6, 4 sets of 4 repetitions at 0.075 kg·m^2^ in Week 7, and 4 sets of 4 repetitions at 0.085 kg·m^2^ in Week 8. The only difference between the two groups was the primary exercise, while all other training components, including warm-up and cool-down exercises, were identical. All repetitions were performed at maximal intended concentric velocity, and participants were instructed to “push or pull as fast and explosively as possible” during each repetition. Two-minute rest intervals were provided between sets. Each session lasted ~25 min, including a 5 min standardized warm-up, a 15 min main workout, and a 5 min cool-down. A certified strength and conditioning coach supervised all sessions and provided standardized cues to encourage maximal effort.

### 2.4. Outcome Measurement

#### 2.4.1. Anthropometrics

Body composition was assessed using a bioelectrical impedance analyzer (InBody 270, Seoul, Republic of Korea). The participants were instructed to remove all metal accessories and clean their hands and feet before stepping onto the device. With hands and feet correctly positioned on the electrodes, the participants stood still, according to the device’s instructions. All tests were performed in the morning before the participants ate in order to reduce the impact of interference factors on the experimental results. The following variables were recorded and used as baseline characteristics: body weight, body fat percentage, and skeletal muscle mass. Height was measured using a stadiometer, with participants standing upright and looking straight ahead. The following variables were recorded and used as baseline characteristics: body mass, body fat percentage, skeletal muscle mass, and height.

#### 2.4.2. Vertical Jump Test

Vertical jump performance was measured using dual force plates (AMTI Inc., Newton, MA, USA) at a sampling rate of 1000 Hz. Two types of jumps were assessed: countermovement jump (CMJ) and squat jump (SJ). Participants held a lightweight bar (<1 kg) across the upper trapezius using a natural grip. They stood upright with each foot placed evenly on separate force plates.

For CMJ, participants following a verbal cue (“3, 2, 1, jump!”). They rapidly descended to a self-selected depth and immediately jumped vertically, with maximal effort. The knees remained extended during flight, and participants landed naturally. After landing, they stood still on the force plate for approximately 2 s before stepping off. Two familiarization trials were performed prior to testing. Three valid trials were collected, with 1 min rest intervals between each. Force–time data were recorded using a force plate. Variables were extracted based on the methods of Cabarkapa et al. [21], and the system weight minus 5 standard deviations was used as the starting point of the movement. The variables were extracted according to the conversion of velocity values, including the eccentric phase, i.e., eccentric duration, peak force, acceleration duration, acceleration impulse, deceleration duration, deceleration impulse; the concentric phase, i.e., propulsion duration, mean force, mean power, and impulse; and overall performance, i.e., jump height, peak force, peak power, modified RSI, and concentric/eccentric impulse ratio.

For SJ, the participants squatted to a 90° knee flexion, verified with a goniometer, and jumped vertically with maximal effort upon command. No additional countermovement was allowed. The knees remained extended during flight, and the landing was natural. After landing, the participants stood still for ~2 s before stepping off the device. Two practice trials were completed. Three test trials were performed, with a 1 min rest interval between trials. Force plate data were used to extract the concentric mean force, mean power, impulse, jump height, peak force, and peak power.

#### 2.4.3. Swimming Start Performance Test

Swimming start performance was assessed in a 25 m short-course pool using an electronic timing system (PT-7000, SEIKO, Tokyo, Japan) and two synchronized video cameras (FX5, Sony, Tokyo, Japan) recording at 60 frames per second. All participants performed a kick-start from a competition block (AWE017 Super Block 800, Anti Wave Global Pty Ltd., Brisbane, Australia) and a front crawl swim. One camera was positioned laterally between the starting block and the 5 m line to capture the start signal and early flight phase, while the second camera was mounted 6 m above the pool’s midline to capture sagittal-plane whole-body movement. The flash signal from the SEIKO system served as the start reference frame. Swimmers were instructed to perform a maximal-effort freestyle start upon noting the electronic signal and swim to 25 m. Each swimmer completed two trials, with a 5 min rest between attempts. The better performance of the two was selected for analysis. Video analysis was conducted using Kinovea (v0.8.26, Paris, France). Calibration was performed using fixed pool markings for scale. The following variables were extracted [6,22]: flight time (FT): time from take-off (toe leaves block) to water entry (fingertip contact); flight distance (FD): horizontal distance from block edge to water entry point (fingertip contact); mean horizontal hip velocity (MHHV): horizontal flight displacement of the hip divided by FT; underwater speed (UWS): distance from water entry to breakout point (any body part breaks surface) divided by time underwater; 15 m swim speed (S15) and 25 m swim speed (S25): distance divided by the time from the start signal to the head crossing the respective lines.

### 2.5. Statistical Analysis

To facilitate interpretation and comparison, both pre- and post-test values are presented as mean ± standard deviation (SD). Additionally, absolute delta values (post–pre) were included in the tables to illustrate the magnitude of change. All inferential statistical analyses were performed using raw (non-normalized) data. Data are presented as mean ± standard deviation (SD). Normality was assessed using the Shapiro–Wilk test, confirming that all variables met the assumption of normal distribution. Baseline comparisons were performed using independent samples *t*-tests to ensure equivalence between groups prior to the intervention. A mixed-design analysis of variance (ANOVA) was conducted to examine the main effects of time (pre vs. post), group (HOR vs. VER), and their effect on the jump and swim performance variables. Bonferroni post hoc corrections were applied, where appropriate. Effect sizes (ES) were calculated based on Cohen’s criteria: <0.2 (trivial), 0.2 (small), 0.4 (moderate), 0.6 (large), 1.2 (very large), and 2.0 (extremely large). Given the relatively small sample size (*n* = 16), Spearman’s rank correlation coefficients were used to explore monotonic relationships between changes in jump metrics and swim start performance. All statistical analyses were conducted using MedCalc (MedCalc Software Ltd., Ostend, Belgium), with a significance level set at α = 0.05.

## 3. Results

### 3.1. Participant Characteristics

Table 1 presents the descriptive characteristics of the study participants. An independent samples t-test was used to compare baseline differences between the HOR and VER groups. No statistically significant differences were observed in age (*p* = 0.595), height (*p* = 0.388), body mass (*p* = 0.247), body fat percentage (*p* = 0.315), lean body mass percentage (*p* = 0.422), training experience (*p* = 0.089), or World Aquatics points (*p* = 0.149). Additionally, there were no significant group differences in baseline swim performance measurements, including 15 m swim speed (*p* = 0.622) and 25 m swim speed (*p* = 0.981).

### 3.2. Swimming Performance

**Figure 2:**
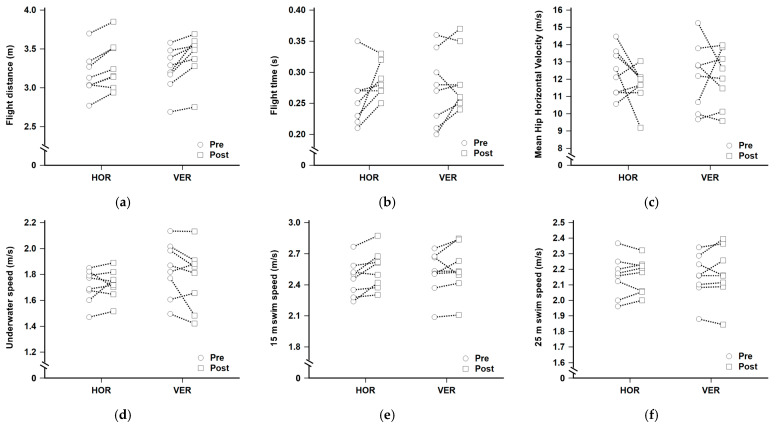
Individual pre- and post-test changes in swim performance variables for each participant: (**a**) flight distance, (**b**) flight time, (**c**) mean horizontal hip velocity, (**d**) underwater speed, (**e**) 15 m swim speed, and (**f**) 25 m swim speed. Note: HOR = horizontal vector training; VER = vertical vector training.

Table 2 summarizes group-level changes in swim start and sprint performance following the intervention. Figure 2 shows individual pre- to post-test values regarding the swim performance variables. The time effects were observed for flight distance (*p* = 0.001, ES = 2.007), flight time (*p* = 0.027, ES = 0.883), and 15 m swim speed (*p* = 0.018, ES = 0.942). No group or interaction effects were found for these variables. No changes were observed in MHHV (*p* = 0.495, ES = 0.249), 25 m swim speed (*p* = 0.394, ES = 0.321), or UWS (*p* = 0.351, ES = 0.321), with no main or interaction effects reported.

### 3.3. Countermovement Jump

Table 3 summarizes the standardized changes in CMJ performance variables following the intervention. In the eccentric phase, no group or time effects were observed across variables. The effect sizes were moderate for eccentric acceleration duration (ES = 0.419) and eccentric deceleration impulse (ES = 0.417). Other eccentric variables showed small or trivial effect sizes. In the concentric phase, a time effect was observed for concentric impulse (*p* = 0.049, ES = 0.720). Concentric mean power increased in the HOR group, with a large effect size (ES = 0.600) and no time effect. Concentric mean force showed minimal change across groups and no time effect (*p* = 0.041, ES = 0.823). For overall jump performance, time effects were found for jump height (*p* = 0.059, ES = 0.614) and peak power (*p* = 0.012, ES = 0.934). No group or interaction effects were observed. Peak force and RSI mod showed no changes and trivial effect sizes.

### 3.4. Squat Jump

Table 4 presents the percentage changes in the squat jump (SJ) performance variables following the eight-week intervention. A time effect was observed for jump height (*p* = 0.029, ES = 0.885) and peak power (*p* = 0.018, ES = 0.976). Concentric impulse also showed a time effect (*p* = 0.026, ES = 0.877), with an increase in the VER group (+7.8%) and a decrease in the HOR group (−3.1%). No group or interaction effects were detected for concentric mean force (*p* = 0.099, ES = 0.645), concentric mean power (*p* = 0.073, ES = 0.709), or peak force (*p* = 0.451, ES = 0.281).

### 3.5. Correlation Between Changes in Countermovement Jump Variables and Swim Performance

Spearman’s correlation analysis was performed using delta scores (post–pre) to evaluate the relationships between changes in CMJ variables and changes in swim performance. As summarized in Table 5, moderate correlations were observed between improvements in concentric phase variables and 15 m swim speed, including concentric mean force (*r* = 0.450, *p* = 0.080), concentric mean power (*r* = 0.476, *p* = 0.062), and concentric impulse (*r* = 0.456, *p* = 0.086). Additionally, eccentric deceleration impulse also showed a moderate correlation with changes in 15 m swim speed (*r* = 0.450, *p* = 0.080). No significant correlations were found between changes in jump variables and 25 m swim speed, MHHV, or UWS. Correlation coefficients for flight distance (FD) and flight time (FT) were generally low to moderate, and none reached statistical significance.

## 4. Discussion

The purpose of this study was to investigate the effects of horizontal and vertical vector training on swim start performance and lower extremity neuromuscular output in collegiate division-one swimmers in Taiwan. Although no significant group effects or interactions were observed, several key performance variables demonstrated the main effects of time, including 15 m swim speed, countermovement jump (CMJ) and squat jump (SJ) concentric performance, and jump height. These findings suggest that both training modalities are effective in improving neuromuscular performance. Furthermore, correlation analyses revealed moderate associations between concentric phase variables and swim start performance. Specifically, 15 m swim speed was positively correlated with CMJ concentric mean power (r = 0.476, *p* = 0.062) and concentric impulse (*r* = 0.456, *p* = 0.076). Although these correlations did not reach traditional levels of statistical significance, they suggest a meaningful relationship between lower limb concentric performance and early phase swimming acceleration.

Previous studies have suggested that horizontal vector training may provide better results than vertical training for tasks involving horizontal displacement, such as sprinting and acceleration [18,23]. Although no statistically significant between-group differences were found in this study, the HOR group improved its 15 m swimming speed by approximately 3%, on average, while the VER group improved by approximately 1%. One potential explanation for the lack of a significant interaction is the technical complexity of the swimming start, which involves the coordinated integration of multiple movement elements. Although the kick-start module was introduced in 2008 [24], swimming start performance remains highly dependent on various technical variables, such as weight distribution strategy (e.g., forward or backward positioning) [25], rear leg drive timing, and arm swing dynamics [3,4,26]. These technical factors may mask the independent effects of horizontal and vertical resistance training, thereby limiting the detection of clear differential responses to training methods. Although some studies have shown gender differences in swimming start mechanics [10], this study did not include enough female participants to thoroughly investigate this aspect. Nonetheless, the performance improvements observed in the HOR group are consistent with previous findings in other sports, such as sprinting and basketball, which have demonstrated that horizontal resistance training can enhance explosive starts and linear acceleration [17,18,23]. Despite the lack of a statistically significant interaction, the results suggest that horizontal training may have equal potential for improve swimming start performance as that of vertical training. Additionally, it is noteworthy that no significant increase in swimming speed beyond 15 m was observed in either training group. This finding may reflect the transition to a clean swimming phase, during which propulsion relies primarily on upper body stroke technique and streamlined hydrodynamic positioning. Therefore, the relative contribution of lower body force production is reduced, which may explain the lack of performance differences at the 25 m distance [20].

After eight weeks of training, significant improvements were observed in concentric force–time characteristics during both CMJ and SJ assessments, particularly in regards to mean force, impulse, and peak power, with large effect sizes reported across multiple variables. These findings align with those in previous literature emphasizing concentric output as a critical predictor of linear acceleration performance [27]. Similarly, the present study suggests that improvements in concentric strength may underlie enhanced 15 m swim start performance. To further support this interpretation, correlation analyses revealed moderate associations between 15 m swim speed and both concentric mean power (*r* = 0.476, *p* = 0.062) and concentric impulse (*r* = 0.456, *p* = 0.076). Although these relationships did not meet conventional thresholds for statistical significance, they highlight a meaningful trend indicating the potential role of lower-limb concentric power production in early-phase swim acceleration. It is worth noting that previous research has suggested that improvements in vertical force characteristics (i.e., VER) may not effectively transfer to swim start performance [13]. Interestingly, none of the eccentric phase variables demonstrated meaningful correlations with swim start performance metrics, further reinforcing the dominant role of concentric mechanics in swim propulsion. These results suggest that, in the context of swim starts, concentric force–time output may play a more critical role than eccentric characteristics—particularly when initiating horizontal movement off the starting block.

The lack of statistically significant interactions may be attributable to several methodological and contextual factors. First, the relatively small sample size (*n* = 16, 8 participants per group) makes it difficult to detect subtle differences between groups in regards to performance adaptations in well-trained collegiate swimmers. Second, the participants were only verbally instructed to avoid coffee and alcohol during the experimental period, and no further testing was performed. Third, since the participants were active athletes, their daily training was not stopped during the experimental period, which may have caused interference effects [28] and weakened the expected results of this study. Fourth, swimming start performance is affected by various highly technical aspects, such as timing and weight distribution strategy, which were not specifically targeted or controlled in this intervention. In this study, participants performed swimming training primarily in the form of ultra-short race-pace training (USRPT) or high intensity interval training (HIIT), which is designed to improve physiological function [29,30], with less emphasis on improving the start technique. Furthermore, due to high training volume, swimmers often perform technique training in a fatigued state, which may limit the effective transfer of land-based strength gains to swimming start performance [31]. In addition, the low proportion of female swimmers (only one in each group) limited our ability to fully explore potential gender differences. Finally, this study did not include a detailed kinematic analysis or a precise technical evaluation of the underwater swimming phase, limiting our understanding of the exact biomechanical mechanisms behind the observed changes.

These findings suggest that both horizontal and vertical vector resistance training can be valuable tools for coaches seeking to enhance swim start performance, as both approaches led to performance improvements. While traditional vertical force exercises remain important, horizontal vector exercises—such as hip thrusts, horizontal jumps, and sled pushes—should also be incorporated to support swim-specific propulsion and lower-body power development. When monitoring or predicting swim start performance, force–time profiling—particularly concentric phase metrics—may provide meaningful insights into an athlete’s progress. Future research should explore longer intervention periods to promote greater neuromuscular adaptations and performance transfer. Training plans should also be integrated within a periodized framework, especially given the potential interference effects caused by the high training volume in swimming. Moreover, incorporating more comprehensive biomechanical analyses, including underwater video and detailed kinematic assessments, may help clarify the mechanisms driving performance gains.

## 5. Conclusions

This study compared the effects of horizontal and vertical vector training on swim start performance and lower-limb neuromuscular output in competitive swimmers. No significant group or interaction effects were found. However, several performance variables showed time-related improvements resulting from both vertical and horizontal vector training. Changes in concentric performance showed a moderate correlation with 15 m swimming speed. These findings suggest that coaches should consider both vertical and horizontal vector exercises in practical training, which may have implications for improving swimmers’ starting program performance.

## Figures and Tables

**Table 1:** Descriptive characteristics of study participants.

Participant	HOR (*n* = 8)	VER (*n* = 8)	Pooled (*n* = 16)	*p*-Value
Male participants	7	7	14	-
Female participants	1	1	2	-
Age (years)	20.3 ± 2.5	20.7 ± 2.2	20.5 ± 2.3	0.595
Height (cm)	174.1 ± 8.6	178.6 ± 11.4	176.3 ± 10	0.388
Body mass (kg)	65.8 ± 7.8	71.9 ± 12	68.8 ± 10.3	0.247
Body fat percentage (%)	12.9 ± 2.3	11.2 ± 3.8	12 ± 3.1	0.315
Lean body mass percentage (%)	49.5 ± 2.1	50.6 ± 3	50 ± 2.6	0.422
Training experience (years)	12 ± 4.1	8.4 ± 3.8	10.2 ± 4.3	0.089
15 m swim speed (m/s)	2.5 ± 0.2	2.5 ± 0.2	2.5 ± 0.2	0.622
25 m swim speed (m/s)	2.2 ± 0.1	2.2 ± 0.1	2.2 ± 0.1	0.981
World Aquatics points	726 ± 17	676 ± 79	699 ± 62	0.149

Note: HOR = horizontal vector training; VER = vertical vector training.

**Table 2:** Changes in swimming performance parameters pre- and post-test for both groups.

Variable	HOR			VER			Effect Size	ANOVA		
	Pre	Post	Δ	Pre	Post	Δ		Group Effects	Time Effects	Interaction Effects
Flight distance (m)	3.16 ± 0.28	3.29 ± 0.31	6.0%	3.23 ± 0.28	3.41 ± 0.29	6.2%	2.007	0.422	**0.001**	0.422
Flight time (s)	0.26 ± 0.04	0.29 ± 0.03	5.0%	0.27 ± 0.06	0.29 ± 0.05	5.5%	0.883	0.443	**0.027**	0.443
MHHV (m/s)	12.4 ± 1.4	11.6 ± 1.1	2.3%	12.1 ± 1.9	12.1 ± 1.6	0.7%	0.249	0.377	0.495	0.377
UWS (m/s)	1.71 ± 0.13	1.72 ± 0.11	1.1%	1.84 ± 0.21	1.77 ± 0.24	−3.7%	0.321	0.107	0.351	0.107
S15 (m/s)	2.46 ± 0.17	2.55 ± 0.19	1.5%	2.51 ± 0.21	2.55 ± 0.24	1.4%	0.942	0.295	**0.018**	0.295
S25 (m/s)	2.15 ± 0.13	2.16 ± 0.11	1.1%	2.16 ± 0.14	2.17 ± 0.17	0.7%	0.321	0.749	0.394	0.749

Note: HOR = horizontal vector training; VER = vertical vector training; MHHV = mean hip horizontal velocity; UWS = underwater swim speed; S15 = 15 m swim speed; S25 = 25 m swim speed. Bolded *p*-values indicate statistical significance (*p* < 0.05).

**Table 3:** Changes in countermovement jump (CMJ) parameters pre- and post-test for both groups.

Variable	HOR			VER			Effect Size	ANOVA		
	Pre	Post	Δ	Pre	Post	Δ		Group Effects	Time Effects	Interaction Effects
Eccentric phase										
ECC-T (s)	0.598 ± 0.09	0.67 ± 0.21	10.7%	0.68 ± 0.12	0.703 ± 0.17	2.8%	0.388	0.487	0.299	0.487
ECC-PF (N)	240.5 ± 104.3	221.7 ± 110.9	−11.4%	287.9 ± 165.4	326.5 ± 217.6	8.9%	0.025	0.177	0.944	0.177
ECC-ACC duration (s)	0.39 ± 0.08	0.45 ± 0.203	17.1%	0.44 ± 0.08	0.47 ± 0.14	6.9%	0.419	0.544	0.265	0.544
ECC-ACC impulse (N·s)	−80.8 ± 17.9	−79.9 ± 17.4	1.2%	−87.2 ± 27	−81.5 ± 28.5	−6.6%	0.379	0.329	0.301	0.329
ECC-DEC duration (s)	0.21 ± 0.03	0.21 ± 0.04	2.3%	0.25 ± 0.07	0.23 ± 0.07	−4.5%	0.149	0.310	0.679	0.310
ECC-DEC impulse (N·s)	80.9 ± 17.8	79.8 ± 17.5	1.1%	87.3 ± 27	81 ± 27.3	−7.0%	0.417	0.305	0.256	0.305
Concentric phase										
Propulsion duration (s)	0.31 ± 0.03	0.31 ± 0.03	0.1%	0.33 ± 0.04	0.32 ± 0.04	−2.9%	0.367	0.677	0.331	0.677
CON-MF(N)	1205 ± 201	1235 ± 176	2.0%	1297 ± 202	1332 ± 210	2.8%	0.823	0.971	**0.041**	0.971
CON-MP (W)	1677 ± 396	1751 ± 310	4.9%	1854 ± 354	1876 ± 357	1.5%	0.600	0.334	0.112	0.334
CON impulse (N·s)	169.2 ± 29.5	177.2 ± 28.2	4.4%	197.3 ± 41.5	197.6 ± 37.4	0.6%	0.720	0.110	**0.049**	0.110
Overall Performance										
Jump height (cm)	33.9 ± 5.9	37.3 ± 4.6	11.4%	37.3 ± 5.8	36.6 ± 5.4	−1.4%	0.614	0.019	0.059	0.019
Peak force (N)	1425 ± 207	1455 ± 204	1.7%	1570 ± 267	1609 ± 277	2.5%	0.831	0.910	**0.039**	0.910
Peak power (W)	3046 ± 586	3261 ± 529	6.6%	3529 ± 844	3545 ± 743	1.4%	0.934	0.063	**0.012**	0.063
RSI mod (%)	0.59 ± 0.105	0.59 ± 0.101	1.1%	0.55 ± 0.06	0.55 ± 0.097	−0.4%	0.012	0.889	0.975	0.889
CON-Imp/ECC-Imp (%)	2.12 ± 0.27	2.26 ± 0.33	5.5%	2.37 ± 0.595	2.61 ± 0.76	9.1%	0.909	0.788	**0.026**	0.788

Note: HOR = horizontal vector training; VER = vertical vector training; ECC-T = eccentric duration; ECC-PF = eccentric peak force; ECC-ACC = eccentric acceleration; ECC-DEC = eccentric deceleration; CON = concentric; CON-MF = concentric mean force; CON-MP = concentric mean power; CON-Imp/ECC-Imp = concentric impulse/eccentric impulse. Bolded *p*-values indicate statistical significance (*p* < 0.05).

**Table 4:** Changes in squat jump (SJ) parameters pre- and post-test for both groups.

Variable	HOR			VER			Effect Size	ANOVA		
	Pre	Post	Δ	Pre	Post	Δ		Group Effects	Time Effects	Interaction Effects
CON-MF(N)	1072 ± 238	1103 ± 313	2.2%	1083 ± 291	953 ± 221	−8.9%	0.645	0.865	0.099	0.865
CON-MP (W)	1214 ± 688	943 ± 747	−19.7%	1069 ± 868	630 ± 803	−24.5%	0.709	0.834	0.073	0.834
CON Impulse (N·s)	164 ± 30	168 ± 25	3.1%	181 ± 39	194 ± 38	7.8%	0.877	0.300	**0.026**	0.300
Jump Height (cm)	31.5 ± 5.5	33 ± 4.1	6.2%	33.1 ± 3.7	34.7 ± 4.3	4.8%	0.885	0.778	**0.029**	0.778
Peak Force (N)	1418 ± 201	1404 ± 177	−0.5%	1613 ± 293	1576 ± 296	−2.3%	0.281	0.617	0.451	0.617
Peak Power (W)	2904 ± 582	2996 ± 487	4.1%	3298 ± 720	3407 ± 699	3.6%	0.976	0.860	**0.018**	0.860

Note: HOR = horizontal vector training; VER = vertical vector training; CON = concentric; CON-MF = concentric mean force; CON-MP = concentric mean power. Bolded *p*-values indicate statistical significance (*p* < 0.05).

**Table 5:** Spearman correlation coefficients between changes in countermovement jump variables and swim performance outcomes.

Variable	FD	FT	MHHV	UWS	S15	S25
Eccentric duration	0.288	−0.031	0.008	−0.391	0.003	−0.103
Eccentric peak force	0.285	0.043	−0.009	−0.385	−0.103	0.147
Eccentric acceleration duration	0.271	−0.022	0.026	0.335	−0.056	−0.097
Eccentric acceleration impulse	0.015	0.109	−0.047	0.244	0.429	−0.065
Eccentric deceleration duration	0.106	0.257	−0.225	−0.306	−0.062	−0.188
Eccentric deceleration impulse	0.197	0.096	−0.026	0.068	0.450 *	−0.009
Propulsion duration	−0.109	0.155	−0.197	−0.429	−0.194	0.141
Concentric mean force	−0.142	0.150	0.209	0.300	0.450 *	−0.013
Concentric mean power	0.218	−0.009	0.082	0.253	0.476 *	−0.029
Concentric impulse	0.114	0.108	−0.050	0.324	0.456 *	−0.015
Jump height	0.019	−0.135	−0.021	0.335	0.265	−0.256
Peak force	−0.009	−0.125	0.188	0.274	0.200	−0.397
Peak power	−0.115	0.117	−0.097	0.371	0.318	−0.256
RSI mod	−0.397	−0.009	−0.056	0.391	0.115	−0.097
Concentric impulse/eccentric impulse	0.050	−0.131	0.106	−0.100	−0.265	−0.018

Note: FD: flight distance; FT: flight time; MHHV: mean horizontal hip velocity; UWS: underwater speed; S15: 15 m swim speed; S25: 25 m swim speed. * *p* < 0.1.

## Data Availability

The data presented in this study are available on request from the corresponding author. The data are not publicly available due to privacy constraints.
